# CDK5: an oncogene or an anti-oncogene: location location location

**DOI:** 10.1186/s12943-023-01895-8

**Published:** 2023-11-23

**Authors:** Kumar Nikhil, Kavita Shah

**Affiliations:** 1 Department of Chemistry, Purdue University Center for Cancer Research, 560 Oval Drive, West Lafayette, IN 47907 USA; 2 Present Address: School of Biotechnology, Kalinga Institute of Industrial Technology, Bhubaneswar, 751024 India

**Keywords:** CDK5, p35, p39, Cancer, CDK

## Abstract

Recent studies have uncovered various physiological functions of CDK5 in many nonneuronal tissues. Upregulation of CDK5 and/or its activator p35 in neurons promotes healthy neuronal functions, but their overexpression in nonneuronal tissues is causally linked to cancer of many origins. This review focuses on the molecular mechanisms by which CDK5 recruits diverse tissue-specific substrates to elicit distinct phenotypes in sixteen different human cancers. The emerging theme suggests that CDK5’s role as an oncogene or anti-oncogene depends upon its subcellular localization. CDK5 mostly acts as an oncogene, but in gastric cancer, it is a tumor suppressor due to its unique nuclear localization. This indicates that CDK5’s access to certain nuclear substrates converts it into an anti-oncogenic kinase. While acting as a bonafide oncogene, CDK5 also activates a few cancer-suppressive pathways in some cancers, presumably due to the mislocalization of nuclear substrates in the cytoplasm. Therefore, directing CDK5 to the nucleus or exporting tumor-suppressive nuclear substrates to the cytoplasm may be promising approaches to combat CDK5-induced oncogenicity, analogous to neurotoxicity triggered by nuclear CDK5. Furthermore, while p35 overexpression is oncogenic, hyperactivation of CDK5 by inducing p25 formation results in apoptosis, which could be exploited to selectively kill cancer cells by dialing up CDK5 activity, instead of inhibiting it. CDK5 thus acts as a molecular rheostat, with different activity levels eliciting distinct functional outcomes. Finally, as CDK5’s role is defined by its substrates, targeting them individually or in conjunction with CDK5 should create potentially valuable new clinical opportunities.

## Introduction

Cyclin-dependent kinases (CDKs) are a family of proline-directed serine/threonine kinases that require binding to cyclins for enzymatic activation. They phosphorylate SP/TP sites with a preference for basic residues on either side [1, 2]. Although Cyclin-dependent kinase-5 (CDK5) is highly homologous to its relatives, it is an inimitable family member because it possesses its own binding partners and regulators and controls a variety of distinctive cellular functions [3]. CDK5 activation occurs upon binding with Cyclin I or noncyclin activators p35 (aka CDK5R1), p39 (aka CDK5R2), or their cleaved counterparts p25 and p29, all of which are exclusive for CDK5 [4–6]. While classical CDKs are inhibited by Y15 phosphorylation, it has no impact on CDK5 activity [7]. Cell cycle inhibitors such as p27KIP1, p57KIP2 and p21CIP1, which inhibit the kinase activities of cyclin-CDK1 and cyclin-CDK2 complexes, have no effect on the p35/p39-CDK5 complex [8, 9]. In contrast, CDK5 is inhibited by binding glutathione S-transferase P (GSTP1) and Cyclin-E (CCNE) [10–12]. Unlike other CDKs, which are mandatory players in the cell cycle, CDK5 is distantly related to the cell cycle machinery and is activated particularly during DNA damage [13]. Instead, CDK5 is most prominently known for its role in the central nervous system, where it controls essential neuronal functions such as cytoskeletal architecture and dynamics, axonal guidance, neuronal migration, cell adhesion, synaptic transmission, membrane trafficking and drug addiction [14]. Low CDK5 activity leads to several neurodevelopmental disorders, and deregulated CDK5 activity is strongly associated with pathological changes in multiple neurodegenerative diseases [11]. Thus, maintaining optimal CDK5 activity is critical for sustaining neuronal homeostasis.

Importantly, recent studies have uncovered that CDK5 and p35 are expressed in the vast majority of nonneuronal tissues, where they regulate various physiological functions, including hematopoietic cell differentiation, immune response, lymphatic valve formation, myogenesis, melanogenesis, insulin levels, cell migration, wound healing, invasion, survival, and angiogenesis. CDK5 deregulation is causatively linked to several diseases, including cancer, senescence, diabetes, immune dysfunction and inflammation [15]. Nevertheless, CDK5’s contribution to these nonneuronal diseases has been largely overlooked due to its atypical nature and its critical role in the CNS. Accumulating evidence indicates that CDK5 is a master regulator of various oncogenic processes, including tumorigenesis, metastasis, angiogenesis, chemoresistance and tumor immunity. Abnormal expression of CDK5 or its activators (particularly p35) due to amplification, mRNA upregulation or their combinations has been found in several human cancers, including cancers of the colon, breast, lung, ovary, prostate and pancreas, as well as melanoma and hematological malignancies. This review focuses on the role of CDK5 outside of the CNS, with particular emphasis on cancer.

## CDK5 deregulation in neurodegenerative diseases

The most predominant CDK5 activators are CDK5R1 and CDK5R2 (aka p35 and p39, respectively), which were initially believed to be CNS specific; however, later studies confirmed widespread expression of p35 in various tissues under both physiological and pathological conditions. P39, an isoform of p35 with 57% amino acid homology, shows more restricted expression in extraneuronal tissues [5]. In contrast, CDK5 is ubiquitously expressed and often is more abundant than either of its activators. Furthermore, while CDK5 is a stable protein, both p35 and p39 have shorter half-lives. The half-life of p35 is 30–60 min, whereas p39 has a relatively longer half-life of ~ 60 min [16]. In addition, monomeric CDK5 is cytosolic, whereas p35 and p39 have myristoylated regions at the N-terminus, which are responsible for their membrane localization. P39 also has a small tail (amino acids 329–366) at the C-terminus, which is absent in p35. This insertion of p39 binds the intracellular protein muskelin, transporting it to the cell periphery, which in turn is essential for its physiological functions. Interestingly, despite the membrane localization of p35 and p39, active CDK5/p35 and CDK5/p39 complexes are also present in the cytoplasm, in addition to the membrane, which is guided by CDK5-mediated phosphorylation of p35 at S8. This event releases p35 from the membrane, resulting in cytoplasmic localization of the active CDK5/p35 complex [17].

In neuronal cells, CDK5 is physiologically activated by transcriptional upregulation of p35 or p39 in response to many factors, including neurotropic factors, cytokines and growth factors. Under neurotoxic conditions, such as β-amyloid, excitotoxicity, oxidative stress, and calcium deregulation, both p35 and p39 are cleaved by calpain to generate p25 and p29, respectively, which have much longer half-lives due to their resistance to ubiquitin-mediated proteolysis [18, 19]. In addition, loss of the N-terminal myristoylated p10 fragment liberates p25 and p29 from peripheral localization, leading to hyperactivation and mislocalization of CDK5-p25 and CDK5-p29 complexes into the cytoplasm and nucleus. These events thus result in constitutive activation of CDK5, which in turn causes hyperphosphorylation of a variety of atypical targets, causing neurotoxicity [20–27].

Cyclin I (CCNI) is another CDK5-specific activator that binds CDK5 in terminally differentiated cells such as neurons and podocytes [6]. Binding of cyclin I to CDK5 prevents cell death by increasing the expression of the antiapoptotic proteins Bcl-2 and Bcl-xL [6]. Interestingly, Cyclin I is highly expressed in cervical cancer, which in turn increases CDK5 expression, causing drug resistance [28].

CDK5 expression levels were found to be upregulated by TP53-induced glycolysis regulatory phosphatase (TIGAR) to promote DNA damage repair [29]. Recently, it was discovered that CDK5 is also a downstream effector of casein kinase 2 (CK2), which activates CDK5 to mediate white matter ischemia [30], and a possible coregulation between CDK5 and Notch signaling [31].

Similarly, CDK5 possesses its own set of negative regulators and is inhibited by cyclin D1, cyclin E, and GSTP1 [10, 32, 33]. Cyclin D1 is believed to inhibit CDK5 by competing with p35 for binding [32], while Cyclin E inactivates CDK5 by sequestering CDK5 away from its activators, p35 and p39 [33]. GSTP1 inhibits CDK5 activity by both competing with p35 and p25 for CDK5 binding and eliminating oxidative stress [10]. GSTP1 is also found to be a CDK5 regulator in various cancer cell lines [10].

## CDK5 deregulation in cancer

CDK5 is not known to be mutated in cancer. However, unlike neurodegenerative diseases, where CDK5 deregulation is predominantly believed to be due to p25 formation, in cancers, it is mainly due to CDK5 and/or p35 (and sometimes p39) overexpression/amplification, although in medullary thyroid carcinoma (MTC), high p25 levels have been reported [34] (Table 1). Accordingly, in breast cancer, prostate cancer, lung cancer, colorectal cancer, melanoma, pituitary adenoma, leukemia, hepatocellular carcinoma (HCC), gliomas and MTC, both CDK5 and p35 levels are increased (Table 1). P35 is transcriptionally upregulated in response to stimuli such as bFGF, IFN-γ, 1,25-dihydroxyvitamin D3, tumor necrosis factor (TNF)-α and TGFβ. In pancreatic tumors, CDK5, p35 and p39 are overexpressed in more than 90%, 94%, and 75%, respectively, predominantly due to genomic amplification [35]. CDK5 and p35 are also highly expressed in pancreatic neuroendocrine tumors [36]. In ovarian cancer, multiple myeloma (MM), and head and neck cancer, only CDK5 upregulation has been reported. In most of the aforementioned cancers, increased CDK5 and/or activator expression matches with poor prognosis, lymph node metastasis, and overall poor survival, while low CDK5 levels correlate with metastasis-free disease [37]. A single-nucleotide polymorphism (SNP) in the CDK5 promoter region is linked to aggressive prostate cancer in the African-American population [38] and to lung cancer in the Korean population [39].

**Table 1:** Expression of CDK5 and its activators in human cancers

No	Cancer Types	CDK5 status	CDK5 activator status	CDK5 localization	CDK5 functions	References
1	Breast cancer	Overexpressed	p35 overexpressed	Cytoplasmic/membranous*	Proliferation, cell survival, EMT and metastasis	[42, 43]
2	Prostate cancer	Overexpressed	p35 overexpressed	Predominantly Cytoplasmic/membranous, little nuclear staining*	Proliferation, migration, invasion, metastasis and neuroendocrine differentiation	[50, 51]
3	Pancreatic cancer	Overexpressed	p35 and p39 overexpressed	Predominantly Cytoplasmic/membranous, little nuclear staining*	Proliferation, tumorigenesis, migration and metastasis	[35, 36]
4	Medullary thyroid cancer	Overexpressed	p35 and p25 overexpressed	Cytoplasmic/membranous*	Proliferation and tumorigenesis	[34]
5	Gastric cancer	Decreased	p35 low	Excluded from nucleus [41]	Inhibits proliferation and metastasis. Acts as cell cycle suppressor	[40, 41, 64]
6	Colorectal cancer	Overexpressed	p35 overexpressed	Cytoplasmic/membranous*	Proliferation, migration and invasion	[66, 67]
7	Glioma	Overexpressed	p35 and p25 overexpressed	Predominantly cytoplasmic with some nuclear staining [68, 69]	Tumorigenesis, migration and invasion	[68, 69]
8	Hepatocellular carcinoma	Overexpressed	p35 overexpressed	Cytoplasmic/membranous/nuclear*	Tumorigenesis, angiogenesis and metastasis	[77, 78]
9	Pituitary adenoma	Overexpressed	p35 overexpressed	Cytoplasmic [86]	Migration and invasion	[85, 86]
10	Lung Cancer	Overexpressed	p35 overexpressed	Cytoplasmic [87, 88]	Tumorigenesis, metastasis, tumor immunity, radio- and chemoresistance	[87, 88]
11	Melanoma	Overexpressed	p35 overexpressed	Cytoplasmic/membranous*	Tumorigenesis, invasion and metastasis	[101, 102]
12	Ovarian cancer	Overexpressed	-	Cytoplasmic and nuclear [105, 106]	Inhibits cell cycle arrest and apoptosis	[105, 106]
13	Medulloblastoma	Overexpressed	p35 and p39 overexpressed	-	Immune evasion	[103]
14	Head and neck cancer	Overexpressed	-	Predominantly cytoplasmic/membranous, some nuclear*	EMT, invasion and metastasis	[108]
15	Leukemia	mRNA upregulated and downregulated	p35 and Cyclin I mRNA upregulated	-	Survival and proliferation	[109–111]
16	Multiple Myeloma	Overexpressed	-	-	Proliferation and drug resistance	[111, 113]

* denotes subcellular localization of CDK5 as reported in The Human Protein Atlas (https://www.proteinatlas.org/). The Human Protein Atlas does not have separate categories for membrane and cytoplasmic localization. Instead, The Human Protein Atlas categorizes them as cytoplasmic/membranous. As CDK5 is not membrane-bound, it should be mainly cytoplasmic when listed as cytoplasmic/membranous

Importantly, in gastric cancer, CDK5 levels are much lower than those in normal gastric tissues, which correlates with decreased patient survival and metastases [40, 41]. This unique function of CDK5 as a tumor suppressor has been attributed to its nuclear localization in these tissues.

### CDK5 acts as an oncogene in breast cancer

**Fig. 1:**
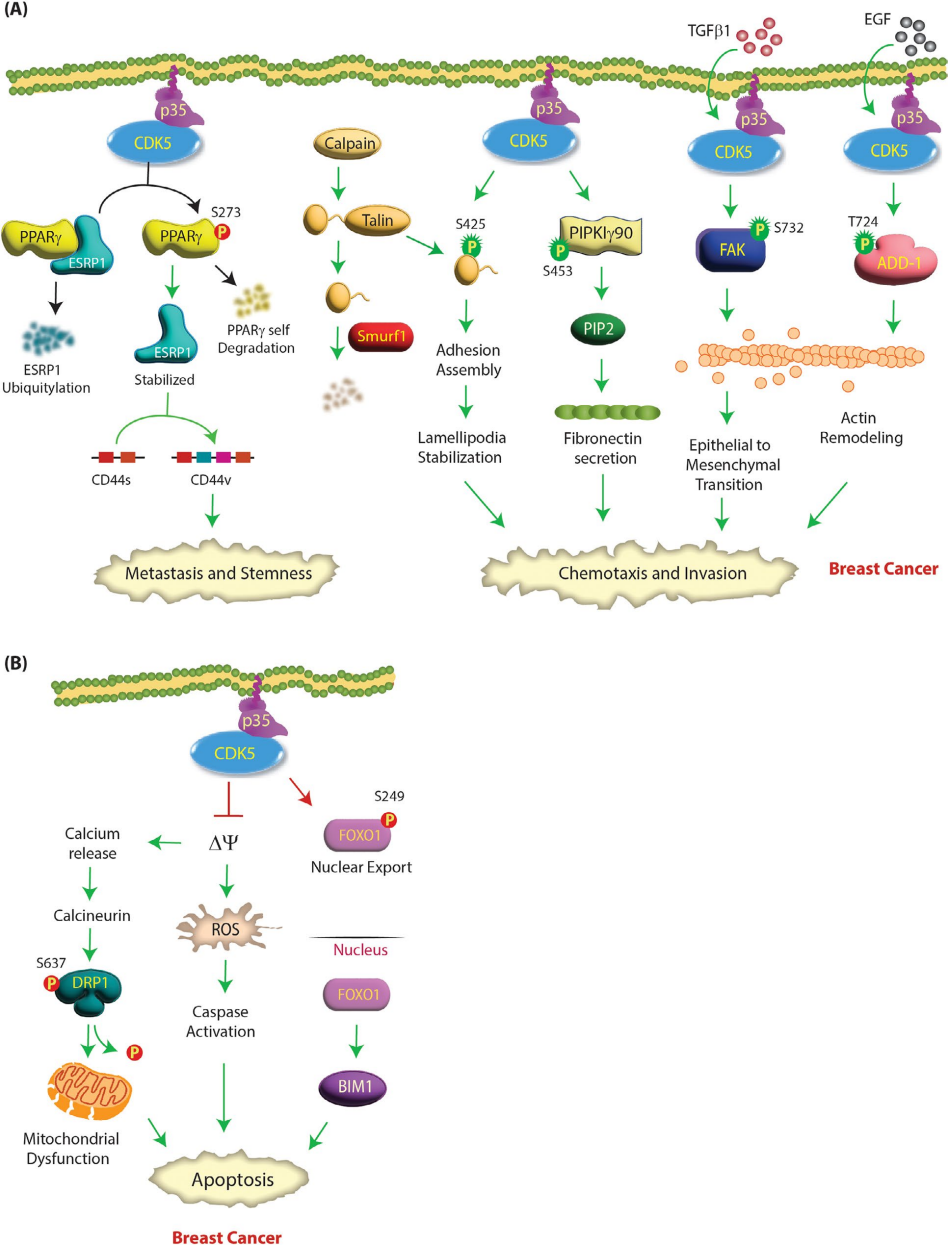
**(A) CDK5 promotes aggressive oncogenic phenotypes in breast cancer by several mechanisms**. The addition of TGF-β1 activates CDK5, which phosphorylates the FAK protein at S732, resulting in changes in the actin cytoskeleton and subsequently leading to EMT and breast cancer progression. CDK5 also promotes the migration of breast cancer cells by directly phosphorylating talin (S425) and PIPKIγ90 (S453). CDK5 is also activated upon EGF stimulation, leading to adducin 1 (ADD1) phosphorylation at T724. This was followed by dynamic remodeling of the actin cytoskeleton, promoting cell migration and invasion in breast cancer. CDK5 phosphorylates PPARγ at S273, which releases ESRP1. ESRP1 is stabilized, and PPARγ is self-degraded. ESRP1 stabilization switches CD44s to the CD44v isoform, which promotes metastasis and stemness. Green and red circles show activating and inactivating phosphorylation events, respectively. **(B) CDK5 promotes aggressive oncogenic phenotypes in breast cancer by inhibiting apoptosis**. CDK5 depletion in breast cancer cells triggers mitochondrial permeability transition pore (mPTP) opening followed by mitochondrial depolarization and an increase in ROS production, which leads to the activation of caspases and cell death. Opening of the mPTP and mitochondrial depolarization also cause calcium release and activation of calcineurin, which causes dephosphorylation of dynamin-related protein 1 (DRP1) at S637, resulting in mitochondrial fragmentation and ultimately cell death. CDK5 knockdown also induces apoptosis in breast tumorospheres by increasing the proapoptotic protein Bim. CDK5 inhibits FOXO1 by phosphorylating it at the S249 site, favoring its nuclear export and inhibiting its transcriptional activity. In the absence of CDK5, nuclear FOXO1 thereafter induces the expression of downstream proapoptotic genes such as Bim, leading to apoptosis

CDK5 and p35 are highly expressed in breast cancer tissues and positively correlate with tumor progression and poor prognosis [42, 43] (Table 1). The mechanism of their upregulation is largely unclear, except for one study demonstrating that transforming growth factor β1 (TGFβ1) stimulation increases their mRNA levels [42]. At the molecular level, active CDK5 facilitates cell migration, metastasis and epithelial to mesenchymal transition (EMT) via actin remodeling, and promotes tumorigenesis by inhibiting apoptotic pathways. TGFβ1 treatment in breast cancer cells triggers active CDK5/p35 to phosphorylate Focal Adhesion Kinase (FAK) at S732, causing metastatic invasion and EMT by potentiating F-actin bundle formation [42] (Fig. 1A). Similarly, Su et al. showed that EGF treatment of MDA-MB-231 cells triggered CDK5-mediated phosphorylation of Adducin-1 (ADD1) at T724, which reduced its ability to bind F-actin (Table 2). ADD1 bundles and caps F-actin barbed ends. Thus, CDK5-mediated phosphorylation exposed the barbed ends, causing elongation and reorganization of the actin cytoskeleton, resulting in cell migration and invasion (Fig. 1A) [44]. Interestingly, the exact mechanism by which EGF activates CDK5 was not explored in this study.

**Table 2:** Direct CDK5 substrates in various cancer types

No	Substrate	Cancer Types	Phosphorylation sites	Protein type	Function	References
1	FAK	Breast cancer	S732	Kinase	Chemotaxis and invasion	[42]
2	PPARγ	Breast cancer	S273	Transcription factor	Metastasis and stemness	[43]
3	ADD1	Breast cancer	T724	Actin-binding protein	Cell migration and invasion	[44]
4	Talin	Breast cancer	S425	Cytoskeletal protein	Migration	[45]
5	PIPKIγ90	Breast cancer	S453	Kinase	Chemotaxis and invasion	[46]
6	FOXO1	Breast cancer	S249	Transcription factor	Inhibits apoptosis	[49]
7	AR	Prostate cancer	S81	Transcription factor	Proliferation	[50]
8	AR	Prostate cancer	S308	Transcription factor	Unknown	[54]
9	STAT3	Prostate cancer	S727	Transcription factor	Proliferation	[55]
10	p21CIP	Prostate cancer	S130	CDK inhibitor	Proliferation	[56]
11	Talin 1 (Talin)	Prostate cancer	S425	Cytoskeletal protein	Invasion	[57]
12	Rb	Prostate cancer	S807, S811	Tumor suppressor	Neuroendocrine Differentiation	[58]
13	EZH2	Pancreatic Cancer	T261	Histone-lysine N-methyltransferase	Inhibits migration and invasion	[61]
14	Rb	MTC	S807, S811	Tumor suppressor	Proliferation	[34]
15	STAT3	MTC	S727	Transcription factor	Proliferation and Tumorigenesis	[62, 63]
16	ERK5	Colorectal cancer	T732	Kinase	Proliferation	[66]
17	CRMP-2	Glioblastoma	S522	Axonal growth and guidance protein	Proliferation	[70]
18	PIKE-A	Glioblastoma	S249, S279	GTPase	Proliferation and migration	[71]
19	TRIM59	Glioblastoma	S308	E3 ubiquitin ligase	Tumorigenesis	[72]
20	Cortactin	Glioblastoma	T145, T219	F-actin-binding protein	Migration	[73]
21	ACSS2	Glioblastoma	S267	Metabolic enzyme	Tumorigenesis	[74]
22	DRP1	Glioblastoma	S616	GTPase	Mitochondrial fission	[76]
23	TPX2	HCC	S486	Spindle assembly factors	Protein stability and metastasis	[80]
23	HIF-1α	HCC	S687	Hypoxia inducible factors	Angiogenesis	[83]
24	PRMT1	HCC	S307	Arginine methyltransferase	Tumorigenesis	[84]
25	VEGFR2	Pituitary Adenoma	S229	Tyrosine kinase receptor	Invasion and metastasis	[86]
26	CAP1	Lung cancer	S308 and S310	Actin-binding protein	Proliferation, migration and invasion	[91, 92]
27	DLC-1	Lung cancer	S120, S205, S422, S509	Tumor suppressor	Inhibits tumorigenesis	[96]
28	Vimentin	Melanoma	S56	Cytoskeletal protein	Invasion and metastasis	[101]
29	NOXA	Leukemia	S13	Pro-apoptotic factor	Proliferation and survival	[112]

Talin is known to be phosphorylated by CDK5 in both breast and prostate cancer. STAT3 and Rb are phosphorylated by CDK5 in prostate cancer and MTC

CDK5 also promotes chemotaxis in breast cancer cells by phosphorylating Talin’s head domain. Calpain cleaves talin, creating a globular head domain and a C-terminal rod domain. The talin head domain binds to Smurf1, an E3 ubiquitin ligase, and is degraded (Fig. 1A). CDK5-mediated phosphorylation of talin at S425 prevents its binding to Smurf1, inhibiting its degradation (Table 2). The talin head domain limits focal adhesion turnover, stabilizing lamellipodia and leading to cell migration [45]. CDK5 phosphorylates phosphatidylinositol 4-phosphate 5-kinase type I γ90 (PIPKIγ90) at S453 (Table 2), which regulates PIPKIγ90 activity to control fibronectin secretion and, consequently, cell invasion [46]. CDK5 mediates stemness in triple-negative breast cancer (TNBC) by phosphorylating Peroxisome proliferator-activated receptor gamma (PPARγ) at S273 (Table 2), which curbs its E3 ligase activity and releases epithelial splicing regulatory protein 1 (ESRP1). As a result, ESRP1 is stabilized, and PPARγ is degraded. ESRP1 promotes CD44s isoform switching to CD44v, which causes stemness transformation and metastasis [43] (Fig. 1A).

CDK5 likewise promotes tumorigenesis by inhibiting cell death pathways. Loss of CDK5 in breast cancer cells opens up the mitochondrial permeability transition pore (mPTP), initiating increased mitochondrial depolarization and higher ROS levels, with subsequent activation of caspases and cell death (Fig. 1B). This is in contrast with AD, where CDK5 hyperactivation results in mitochondrial dysfunction resulting in neuronal death [47]. In tandem, mitochondrial depolarization also promotes calcium release, causing calcineurin activation, which dephosphorylates dynamin-related protein 1 (DRP1) at S637. DRP1 dephosphorylation triggers mitochondrial fragmentation, resulting in cell death [48]. Similarly, CDK5 inhibition or knockdown induces apoptosis in tumor spheres by stabilizing the transcription factor FOXO1. This results in increased levels of the proapoptotic protein Bim1 and decreased in vivo tumor volume [49] (Fig. 1B).

### Role of CDK5 in prostate cancer (PCa)

Similar to breast cancer cells, prostate cancer cells express high levels of CDK5 and p35 (Table 1) [50, 51]. Thus, CDK5 is frequently deregulated in prostate cancer, and its levels strongly correlate with poor clinical prognosis. CDK5 promotes cell proliferation and invasion in PCa.

**Fig. 2:**
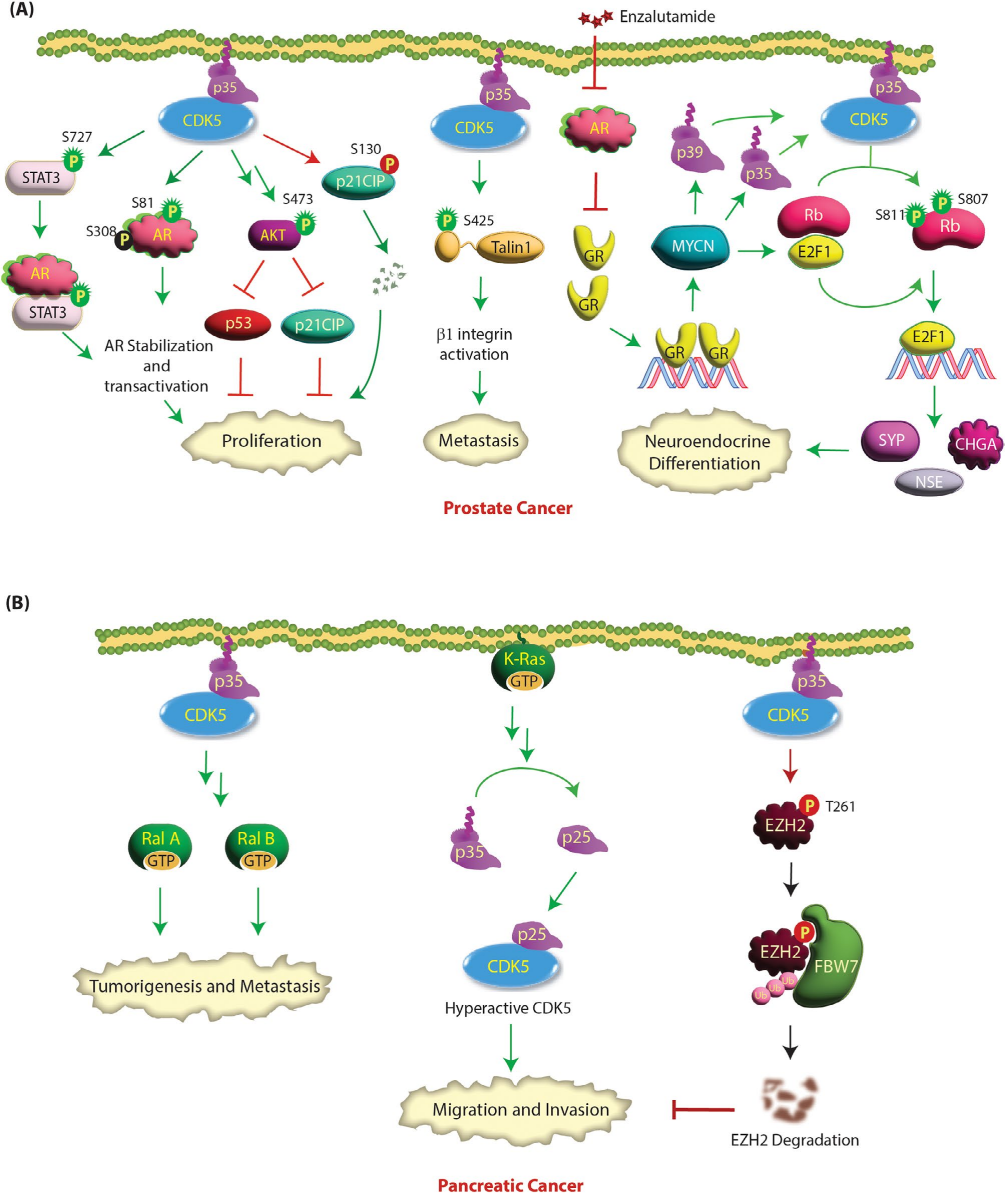
**(A) CDK5 facilitates highly oncogenic phenotypes in prostate cancer**. CDK5 can simultaneously affect numerous targets and promote PCa progression. CDK5/p35 overexpression in prostate tumor cells increases the phosphorylation of talin1 at S425, which induces a conformational change resulting in talin1 activation. Phosphorylated talin1 then binds to the cytoplasmic tail of β1 integrin, leading to β1 integrin activation and downstream integrin signaling. This leads to increased cell survival, adhesion, motility and metastatic potential of PCa cells. CDK5 also activates STAT3 and AR proteins by phosphorylation at specific sites. Activation of STAT3 or AR causes prostate cancer proliferation. CDK5 indirectly activates AKT resulting in p53 and p21CIP downregulation and cell cycle progression. CDK5 directly phosphorylates p21CIP at S130, which degrades it, promoting oncogenesis. Enzalutamide treatment upregulates GR signaling, which transcriptionally increases MYCN levels. MYCN increases p35, p39 and E2F1 transcription. p35 and p39 binding activates CDK5, which phosphorylates Rb at S807 and S811, releasing E2F1, which in turn transcriptionally upregulates the NEPC genes, leading to neuroendocrine differentiation. Green and red circles show activating and inactivating phosphorylation events, respectively. Green and red arrows represent activating and inactivating pathways, respectively. **(B) CDK5 signaling in pancreatic cancer.** K-Ras activates CDK5 by promoting the cleavage of p35 to p25, although the exact mechanism is not known. CDK5 induces cell migration and invasion through activation of RalA and RalB in pancreatic cancer. CDK5 directly phosphorylates EZH2 at T261, causing its ubiquitylation by FBW7, which in turn inhibits pancreatic cancer cell migration and invasion

Androgen receptor (AR) is the key driver in PCa pathogenesis. In healthy prostatic epithelial tissues, AR plays an essential role in regulating terminal differentiation, apoptosis suppression and hormone secretion [52]. However, AR signaling is highly deregulated in PCa and facilitates cell proliferation, survival, and invasion during PCa development [53]. CDK5 promotes prostate cancer growth by both activating and stabilizing AR either directly or indirectly [50, 54, 55]. Hsu et al. demonstrated that CDK5 phosphorylates AR at S81 in vitro and in vivo [50] (Table 2). This stabilizes AR, causing enhanced nuclear translocation and activation. Furthermore, AR protein levels showed a significant correlation with CDK5 or p35 in 177 AR-positive PCa patients, underscoring the clinical significance of the CDK5-AR axis in PCa progression [50]. CDK5 also indirectly stabilizes and activates AR by directly phosphorylating STAT3 at S727 in cells and xenograft tumors [55]. Phosphorylated STAT3 interacts with AR, which upregulates AR protein stability and transactivation, enhancing cell proliferation. The authors further provided clinical evidence that the level of p-Ser^727^-STAT3 significantly correlated with Gleason score and CDK5, p35 and AR levels [55] (Fig. 2A).

In contrast, Lindqvist et al. demonstrated that CDK5 phosphorylates AR at S308; however, the consequences of this phosphorylation event on AR stability or transactivation were not analyzed [54]. Nevertheless, this study confirmed that CDK5 knockdown indeed decreases AR stability. The authors further showed that CDK5’s major growth-promoting activity stemmed from its activation of the AKT pathway, although the exact mechanism leading to AKT phosphorylation at S473 remained unclear [54]. AKT activation downregulated p21CIP1 and p53 proteins, allowing cell cycle progression. Huang et al. further explored the link between CDK5 and p21CIP1 and showed that CDK5 directly phosphorylates p21CIP1 at S130, which degrades it, thereby releasing CDK2, which promotes oncogenesis [56] (Fig. 2A).

Similar to breast cancer, CDK5 activity is required to control cell motility and the metastatic potential of prostate cancer cells [51]. CDK5 knockdown or pharmacological inhibition resulted in cytoskeletal remodeling and loss of motility and invasiveness in the highly metastatic AT6.3 prostate cancer cell line. Similarly, dominant-negative CDK5-expressing xenografts revealed significantly fewer metastases than controls [51]. Talin1 was also shown to be phosphorylated by CDK5 in prostate cancer cells, which activates β1 integrin, promoting invasion [57] (Fig. 2A).

A recent study revealed CDK5 as a key player that promotes neuroendocrine phenotypes in a prostate cancer patient–derived xenograft (PDX) treatment model upon ADT therapy [58]. Accordingly, enzalutamide treatment in the PDX133-4 model upregulated glucocorticoid receptor (GR) signaling, which transcriptionally increased MYCN levels. MYCN upregulated p35, p39 and E2F1 transcription. P35 and p39 activated CDK5, which directly phosphorylated Rb at S807 and S811, releasing E2F1, which in turn transcriptionally upregulated the NEPC genes synaptophysin (SYP), chromogranin A (CHGA) and neuron-specific enolase (NSE), leading to neuroendocrine differentiation (Fig. 2A) [58]. This study revealed CDK5 as a potential target for NEPC.

### CDK5 acts as an oncogene in pancreatic cancer

CDK5, p35 and p39 are overexpressed in more than 90%, 94%, and 75% of pancreatic ductal adenocarcinoma (PDAC) tumors, respectively, predominantly due to genomic amplification (Table 1) [35]. As noted before, CDK5 and p35 are also abundantly expressed in pancreatic neuroendocrine tumors [36]. Furthermore, CDK5 is also hyperactivated in pancreatic cancer due to mutant K-Ras. K-Ras activating mutations are hallmarks of pancreatic cancer. K-Ras hyperactivates CDK5 by cleaving p35 into p25, promoting increased cell migration and invasion [35]. However, the exact mechanism by which K-Ras promotes p25 formation remains unknown. CDK5 ablation inhibited orthotopic tumor formation and systemic metastasis in vivo [59] (Fig. 2B).

Interestingly, in pancreatic cancer, Ral guanine nucleotide-exchange factors (Ral GEFs) have emerged as the key effectors of the K-Ras pathway instead of the B-Raf, extracellular signal-regulated kinase (ERK) or MAPK pathways. As Ral GEFs activate RalA and RalB small G proteins, Lim et al. demonstrated that RalA is critical for Ras-driven tumor initiation and that RalB is crucial for Ras-driven tumor metastasis [60]. As CDK5 is also critical for pancreatic cancer progression, Feldmann et al. investigated a potential link between CDK5 and RalA/B GTPases [59]. Using KRAS2 mutants, they reported that the loss of RalA function inhibits the tumorigenicity of pancreatic cancer cells, which is dependent on CDK5. Accordingly, dominant-negative CDK5-expressing cells exhibited decreased levels of active RalA-GTP and RalB-GTP. Furthermore, constitutively active forms of RalA or RalB could overcome the effect of dominant-negative CDK5, suggesting that Ral proteins are downstream of CDK5. Although the exact molecular mechanism was not delineated, CDK5 may regulate the activation of RalA and RalB by controlling the function of either RalGEFs or RalGAP, suggesting CDK5 as a possible target for pancreatic cancer [59] (Fig. 2B).

In pancreatic cancer, active CDK5 phosphorylates enhancer of zeste homolog 2 (EZH2), which triggers its degradation via FBW7, a component of ubiquitin ligase (Table 2). As EZH2 degradation inhibits tumor migration and invasion [61], CDK5 acts as a tumor suppressor in pancreatic cancer (Fig. 2B). Nevertheless, as many studies have emphatically shown that CDK5 is critical for pancreatic cancer progression, the clinical significance of CDK5-mediated EZH2 degradation needs to be explored further.

### CDK5 promotes medullary thyroid cancer (MTC)

**Fig. 3:**
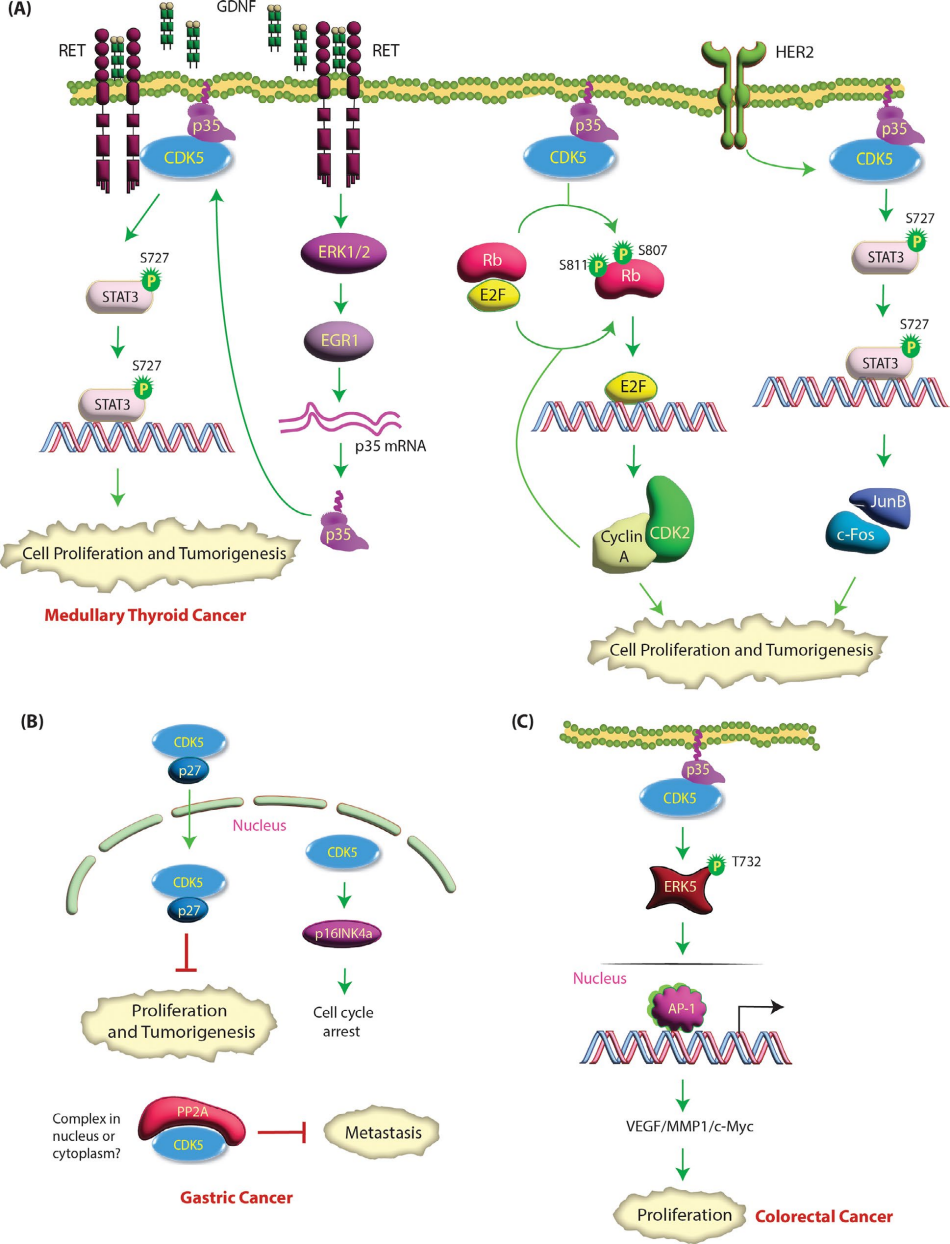
**(A) CDK5 signaling in MTC.** Upon Rb phosphorylation by CDK5, E2F is released and activates the transcription of target genes, including CDK2 and Cyclin A, that mediate cell proliferation. CDK2-Cyclin A further phosphorylates Rb in a positive feedback loop. HER2 signaling activates CDK5 by an unknown mechanism, which in turn phosphorylates STAT3 at S727, promoting cell proliferation and tumorigenesis through cFos-JunB signaling. GDNF activates RET signaling leading to ERK-EGR1 activation, which increases p35 transcription, increasing CDK5 activation. Active CDK5 phosphorylates STAT3 at S727, promoting tumorigenesis. Green circles show activating phosphorylation events. **(B) CDK5-mediated signaling pathways in gastric and colorectal cancer.** In gastric cancer, nuclear CDK5 overexpression inhibits the proliferation and metastasis of gastric cancer cells. Nuclear CDK5 upregulates the CDK inhibitor p16INK4a, which inhibits the S-G2 phase transition, leading to cell cycle arrest. CDK5 binds with p27, which results in its nuclear translocation. CDK5 also binds PP2A, which inhibits metastasis; however, it is unknown whether this event occurs in the cytoplasm and/or nucleus. **(C) CDK5 modulates the ERK5–AP-1 axis to regulate the oncogenic pathway in colorectal cancer.** CDK5 directly interacts with ERK5 and phosphorylates it at T732, thus upregulating the expression of AP-1 and some of its target genes (VEGFA, MMP1 and c-myc). This event results in the malignant development of human CRC both in vitro and in vivo

MTC, although slow-growing, is a highly deadly cancer, partly because it is often diagnosed at an advanced metastatic stage. While thyroid cancers originate from thyroid follicular cells, which produce thyroid hormone, MTC stems from neuroendocrine parafollicular C cells (aka C cells), which synthesize calcitonin. MTC accounts for ~ 1–2% of all thyroid cancers and can be sporadic (75% MTC cases) or hereditary (25%). CDK5 and its activators are present in normal human thyroid tissues but overexpressed in hereditary and sporadic MTC clinical specimens (Table 1) [34]. Pozo et al. showed that inducible expression of p25 in thyroid C cells in vivo leads to MTC, while preventing p25 overexpression inhibits tumor growth [34]. Similarly, targeting CDK5 inhibits sporadic MTC patient-derived cell proliferation. Mechanistically, they showed that active CDK5 phosphorylates retinoblastoma (Rb) protein at S807/811, leading to the expression of CDK2 and cyclin A, which results in cell proliferation (Table 2). Thus, MTC cells overexpressing dominant negative, kinase-dead CDK5 or subjected to p35 knockdown prevented Rb phosphorylation and decreased CDK2 and Cyclin A expression, causing cell cycle arrest (Fig. 3A) [34].

As observed in prostate cancer, CDK5 also phosphorylates STAT3 at S727 in MTC cells. However, contrary to PCa, in which phospho-STAT stabilized AR causing proliferation, in MTC, phospho-STAT3 led to increased cell proliferation and tumor formation via the downstream genes c-*FOS* and *JUNB*. Accordingly, phospho-dead STAT3 (S727A) prevented p35-induced human TT and mouse MTC-M cell proliferation (Fig. 3A) [62]. Subsequent studies revealed the mechanism of CDK5 activation in MTC cells. Upon GDNF stimulation, CDK5 physically interacts with Rearranged-during-transfection (RET) kinase, which activates CDK5 by increasing p35 expression via the ERK1/2-EGR1 pathway [63]. Active CDK5 phosphorylates STAT3 at S727 to promote human medullary cancer cell growth. As activating germline mutations of RET are observed in 100% hereditary and ~ 40% sporadic MTC, CDK5 activation by RET could have profound clinical consequences (Fig. 3A).

### Role of CDK5 in gastric cancer- nuclear CDK5 acts as a Tumor suppressor

Unlike most other cancers, CDK5 acts as a tumor suppressor in gastric cancer. Consequently, CDK5 expression levels are significantly lower in the majority of gastric tumors than in normal gastric tissues, which correlates with decreased patient survival and metastases (Table 1) [40, 41, 64]. Equally importantly, CDK5’s tumor-suppressive functions are mainly attributed to its nuclear localization in these tissues. Cao et al. demonstrated that CDK5 is both nuclear and cytoplasmic in non-tumor tissues. However, it is completely excluded from the nucleus of tumor cells, indicating that nuclear CDK5 acts as anti-oncogene [40]. CDK5 has no intrinsic nuclear localization signal but relies on its binding to p27 using its N-terminal residues (N-17) to translocate to the nucleus [41]. Accordingly, patients with lower levels of CDK5 and p27 display poorer survival compared to patients with either high CDK5 or high p27 or both [41].

Interestingly, CDK5 possesses two nuclear export signals (NES) that bind with the nuclear export mediator CRM-1 and result in cytoplasmic residence of CDK5 [64]. Cao et al. showed that the small molecule NS-0011 increases CDK5 accumulation in the nucleus by disrupting its binding with CRM-1, which in turn suppresses both cancer cell proliferation and xenograft tumorigenesis [40]. Similarly, exogenous expression of nuclear CDK5 inhibits the proliferation of tumor cells and xenografts in nude mice. Overall, these results underscore that CDK5’s nuclear residence drives its function as a tumor suppressor. However, the molecular mechanisms by which nuclear CDK5 acts as a tumor suppressor are largely unknown in gastric cancer, except for one study that showed that nuclear CDK5 upregulates the levels of the CDK inhibitor p16INK4a, which contributes to cell cycle arrest [40] (Fig. 3B).

Lu et al. further revealed that downregulation of CDK5 promotes metastasis in gastric cancer cells, which depends on its interaction with PP2A, a serine/threonine phosphatase [65]; however, this study did not analyze whether PP2A binds cytoplasmic and/or nuclear CDK5. Similarly, the impact of PP2A binding on CDK5 activity or levels was not examined. Nevertheless, similar to CDK5, PP2A is also downregulated in gastric cancer, which is associated with poorer overall survival [65]. Together, these studies demonstrate that CDK5 acts as a tumor suppressor in gastric cancer, likely due to its nuclear localization.

### Role of CDK5 in Colorectal cancer (CRC)

CDK5 and p35 are broadly expressed in human colorectal cancer (CRC) cell lines and human CRC tissues compared to paired normal tissues and cell lines (Table 1) [66, 67]. Higher levels of CDK5 in CRC correlate with advanced disease stage, poor differentiation, increased tumor size and poor prognosis [67]. Likewise, CDK5 kinase activity is also higher in aggressive cell lines such as HCT116 and SW480 compared to less aggressive cell lines such as Caco-2 and Lovo [66]. Similar to other cancers, CDK5 enhances both proliferation and metastasis in CRC [66, 67]. At the molecular level, CDK5 upregulates oncogenic ERK5-AP-1 (activating protein-1) signaling in CRC cells. CDK5 phosphorylates ERK5 at T732, which not only activates its transcriptional activity but also allows ERK5 to act as a transcriptional coactivator for the AP-1 transcription factor. AP-1 is a dimeric transcription factor composed of Jun, Fos, activating transcription factor (ATF) and MAF subunits, all of which bind to common AP-1 binding sites on DNA (Fig. 3C). AP-1 plays a critical role in tumor promotion by increasing the transcription of many crucial oncogenes, including VEGFA, MMP1, and c-myc. Thus, activation of the ERK5–AP-1 pathway by CDK5 increases the expression of VEGFA, MMP1 and c-myc, resulting in human CRC malignancy in vitro and in vivo [66].

### CDK5 is an oncogene in glioblastoma (GBM)

Gliomas or GBM are the most common and lethal malignant brain cancer with high recurrence rates. CDK5 and p35 are overexpressed in gliomas compared to normal brain tissues (Table 1) [68, 69]. Using 152 glioma tissues and 16 normal brain tissues, Yushan et al. reported that CDK5 levels positively correlated with both pathological grade and the Ki-67 labeling index [68]. Catania et al. revealed that CDK5, p35 and p25 are highly expressed in GBM cells and primary tumors and are predominantly cytoplasmic with some nuclear staining. Furthermore, CDK5 staining was constantly higher in grades II to IV astrocytomas as compared to grade I [69]. CDK5 immunoreactivity was non-existent in normal glial cells present in axonal white matter and neuronal grey matter [69]. Equally importantly, they reported that p35 was predominantly present as p25 in 6 out of 7 tumors that were analyzed [69].

**Fig. 4:**
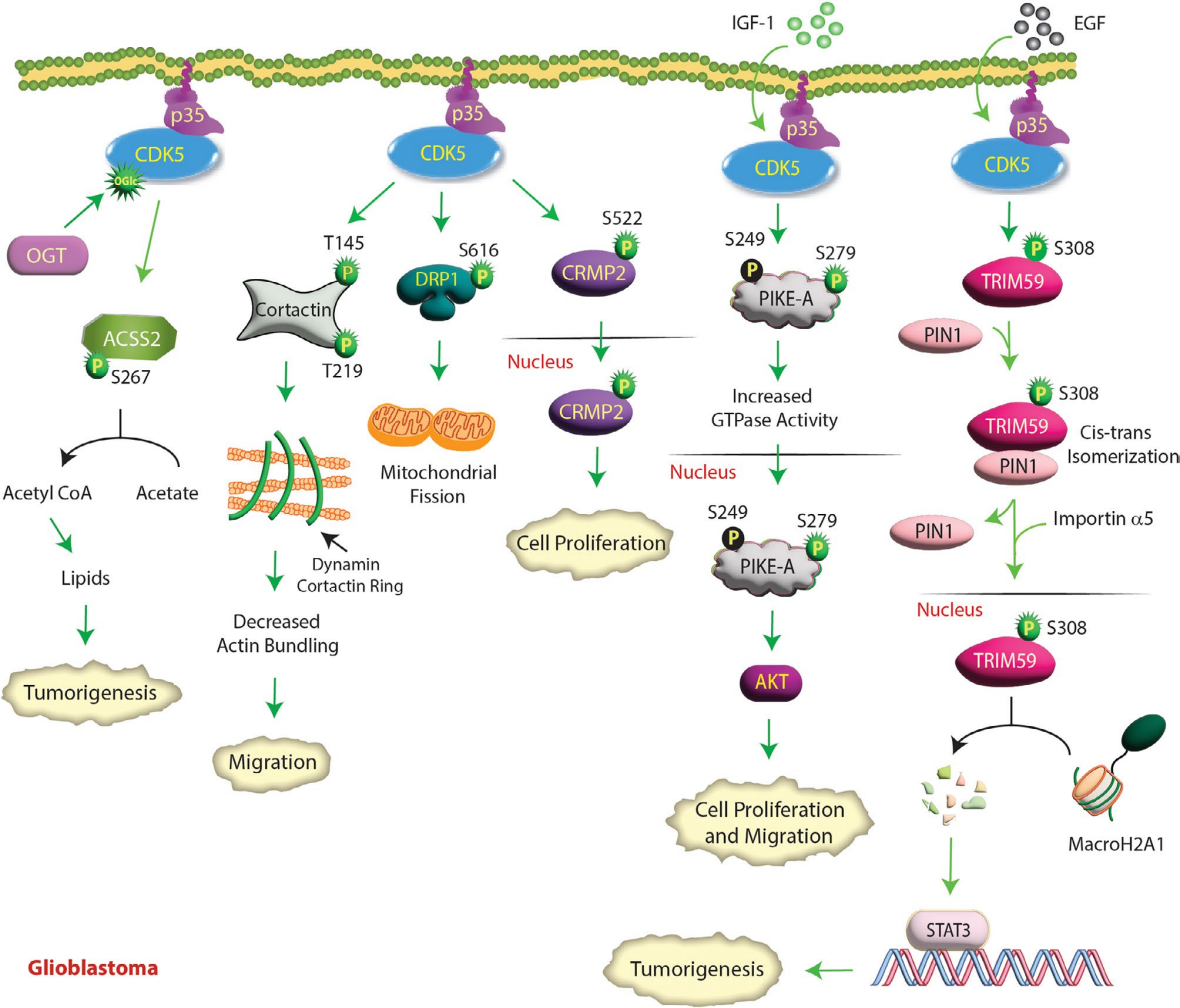
**CDK5 promotes GBM by several mechanisms.** In response to IGF-1, CDK5 phosphorylates PIKE-A at two different sites and stimulates its GTPase activity, which activates nuclear AKT and promotes glioblastoma cell proliferation and migration. Similarly, EGFR-activated CDK5 phosphorylates TRIM59 at S308, which recruits PIN1 for cis–trans isomerization of TRIM59, leading to TRIM59 binding to importin α5 and nuclear translocation. Nuclear TRIM59 enhances STAT3 signaling and tumorigenicity by promoting macroH2A1 ubiquitination and degradation. CDK5-mediated CRMP2 phosphorylation at S522 is another important event that drives glioblastoma cell proliferation. In addition, CDK5 phosphorylates cortactin, which decreases the actin-bundling activity of the dynamin1-cortactin complex, resulting in enhanced cell migration. OGT activates CDK5 by O-GlcNAcylation (OGlc), which in turn phosphorylates acetyl-CoA synthetase 2 (ACSS2) at S267, inhibiting its degradation in GBM cells. ACSS2 converts acetate to acetyl-CoA, which in turn supports tumorigenesis. CDK5 phosphorylates DRP1 at S616, activating it and leading to increased mitochondrial fission

Multiple studies have shown that CDK5 increases proliferation [70] and invasion [71] in glioma cells and tumorigenesis in vivo [72]. Several downstream targets of CDK5 have been identified, which have uncovered the molecular mechanisms by which CDK5 contributes to GBM pathology. In response to insulin growth factor-1 (IGF-1) stimulation, CDK5 phosphorylates phosphatidylinositol 3 (PI3)-kinase enhancer-A (PIKE-A) at the S249 and S279 sites (Table 2). However, S279 phosphorylation is the primary site that activates PIKE-A GTPase activity and triggers its nuclear translocation (Fig. 4). Nuclear PIKE-A activated AKT and promoted glioblastoma cell migration and invasion [71]. The PIKE-A gene is amplified in neuroblastoma cells and upregulates highly aggressive phenotypes by directly regulating AKT.

Glioblastoma cell proliferation was also dependent on collapsin response mediator protein 2 (CRMP2) phosphorylation. CDK5 phosphorylates CRMP2 at S522, triggering nuclear translocation and causing cell proliferation (Table 2) [70] (Fig. 4). Neurofibromin binds CRMP2, which protects it from CDK5-mediated phosphorylation. As deletion or mutation of the Nf1 gene, which encodes neurofibromin, is associated with poor prognosis in glioma, it underscores the role of CDK5 in promoting glioma via CRMP2.

CDK5 also regulates glioma tumorigenicity by phosphorylating Tripartite motif containing 59 (TRIM59), a ubiquitin ligase. Upon EGF stimulation, CDK5 phosphorylates TRIM59 at S308 and promotes its nuclear translocation via Peptidylprolyl Cis/Trans Isomerase, NIMA-Interacting 1 (PIN1). PIN1 triggers the cis-trans isomerization of TRIM59, which causes it to bind importin α5, resulting in its nuclear translocation. Nuclear TRIM59 then promotes ubiquitination and degradation of the tumor-suppressive histone variant macroH2A1, causing STAT3 activation and tumorigenicity (Fig. 4) [72]. Additionally, cortactin was also identified as a direct substrate of CDK5. Dynamin and cortactin form a ring‑like complex that bundles and stabilizes actin filaments. Phosphorylation of cortactin by CDK5 at T145/T219 decreased the actin-bundling activity of the dynamin1-cortactin complex, resulting in aberrant lamellipodia and short filopodia, ultimately leading to cell migration [73] (Fig. 4).

GBMs preferentially use acetyl-CoA to fuel tumor growth. As a result, GBMs upregulate O-GlcNAc transferase (OGT), which upregulates acetate conversion to acetyl-CoA and lipids via CDK5 and Acyl-CoA Synthetase Short Chain Family Member 2 (ACSS2). OGT-mediated O-GlcNAcylation increases CDK5 activity, which phosphorylates ACSS2 at S267, inhibiting its degradation and promoting the synthesis of acetyl-CoA from acetate [74]. ACSS2 phosphorylation is critical for OGT-mediated GBM growth in vitro and in vivo. Thus, the OGT/CDK5/ACSS2 axis directly links CDK5 to metabolic dependencies in GBM (Fig. 4).

Glioblastoma exhibits striking tumor heterogeneity leading to cellular hierarchies with self-renewing glioblastoma stem cells (GSCs), also known as brain tumor initiating cells (BTICs), at the apex. GSCs or BITCs are critical drivers of tumor initiation, progression and therapeutic resistance. Dual-specificity tyrosine phosphorylation-regulated kinase 1 A (DYRK1A) promotes differentiation and inhibits stemness acquisition of GSCs by inhibiting CDK5. Although the exact mechanism remains unknown, DYRK1A inhibition or depletion upregulates the CDK5 activators p35 and p39. This causes CDK5 activation, which in turn enhances SOX2 expression, leading to stemness [75]. Xie et al. showed that CDK5 phosphorylates Dynamin-related protein-1 (DRP1) at S616, activating it and leading to increased mitochondrial fission (Table 2) [76]. Although total DRP1 levels are similar in normal and GBM tissues, phospho-S616-DRP1 was selectively increased in tumor tissues, which was associated with poor survival in GBM patients. Similarly, higher CDK5 expression showed a significant correlation with shorter patient survival (Fig. 4).

Interestingly, Catalina et al. observed that a small subset of GBM tumor sections with high CDK5 and p35/p25 levels exhibited apoptotic features. This prompted them to irradiate GBM cells, which did not alter CDK5 levels, but significantly increased p25 levels. Increased p25 formation strongly correlated with enhanced apoptosis [69], indicating that while high CDK5/p35 promotes glioma tumorigenesis, intense CDK5 activity due to p25 formation results in cell death.

### Role of CDK5 in hepatocellular carcinoma (HCC)

HCC is the third leading cause of cancer-related deaths worldwide. Using a large cohort of samples, Zhang et al. showed that CDK5 expression levels were significantly higher in HCC tissues than in normal, cirrhotic, and adjacent noncancerous liver tissues. Furthermore, CDK5 upregulation revealed a positive correlation with metastasis, vascular invasion, differentiation, advanced clinical stages, decreased survival and greater tumor recurrence (Table 1) [77, 78]. Similarly, CDK5 and p35 levels are higher in human HCC/hepatoblastoma cell lines than in primary human hepatocytes.

**Fig. 5:**
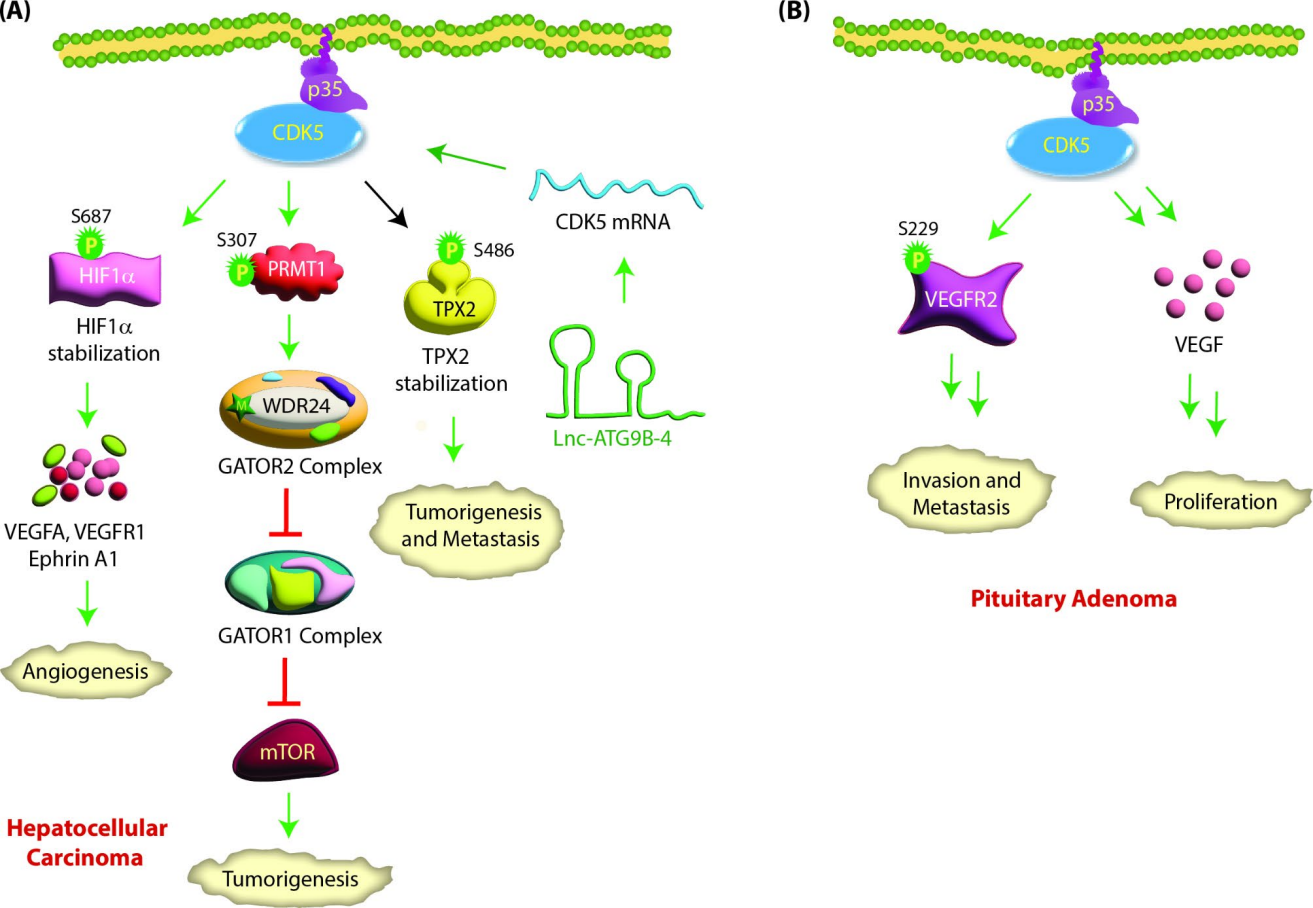
**(A) CDK5 signaling in hepatocellular carcinoma.** CDK5 mRNA levels are upregulated by lnc-ATG9B-4, leading to aggressive phenotypes. CDK5 interacts with hypoxia-inducing factor-1α (HIF-1α) and phosphorylates it at S687. This stabilizes HIF-1α by preventing its proteasomal degradation and promoting angiogenesis by transcriptionally upregulating VEGFA, VEGFR1 and Ephrin A1. CDK5 also regulates metastasis in HCC by regulating TPX2. CDK5-mediated phosphorylation of TPX2 at S486 increases its stability, leading to tumorigenesis and metastasis. CDK5 activates mTORC1 by phosphorylating PRMT1 at S307, which results in its cytoplasmic translocation. Active PRMT1 in turn methylates WDR24, a critical component of the GATOR2 complex. This results in the inhibition of GATOR1, which in turn activates mTOR signaling, leading to tumorigenesis. **(B) CDK5 promotes pituitary adenoma**: CDK5 increases VEGF expression in pituitary adenoma. CDK5 also associates with VEGFR2 and phosphorylates it at S229, which is required for normal cell surface expression of VEGFR2 and for inducing cell migration and invasion

Lnc-ATG9B-4, a long noncoding RNA, is overexpressed in hepatic cancer and is strongly associated with tumor size, TNM stage, portal vein tumor thrombus, metastasis and poor prognosis. Li et al. showed that CDK5 mRNA levels are upregulated by lnc-ATG9B-4 in hepatic cancer, resulting in aggravated oncogenic phenotypes [79] (Fig. 5A).

CDK5 increases proliferation, migration and invasion in HCC by stabilizing TPX2 via S486 phosphorylation (Table 2) [80]. TPX2 is a prognostic marker for HCC and contributes to tumorigenesis, EMT and metastasis [81, 82]. CDK5 promotes angiogenesis in vitro and in vivo by phosphorylating HIF-1α at S687, which stabilizes it. HIF-1α promotes blood vessel formation via transcriptional upregulation of VEGFA, VEGFR1 and EphrinA1 [83]. CDK5 also activates mTORC1 signaling in response to amino acids [84]. mTORC1 senses amino acid signals via the GATOR2 complex. In response to amino acids, CDK5 phosphorylates protein arginine methyltransferase 1 (PRMT1) at S307, which triggers nuclear PRMT1 to translocate to the cytoplasm and lysosome. Cytoplasmic PRMT1 methylates WDR24, an essential component of GATOR2, to activate the mTORC1 pathway, leading to tumorigenesis (Fig. 5A).

### Role of CDK5 in pituitary adenoma

Pituitary adenomas are monoclonal adenomas that constitute approximately 15–20% of intracranial tumors. CDK5 expression and activity are significantly higher in invasive pituitary adenomas than in noninvasive pituitary adenomas. In addition, p35 mRNA and protein levels are also higher in pituitary adenomas than in normal glands (Table 1) [85, 86]. VEGF and its receptors are widely expressed in pituitary tumors and critical for CDK5-mediated cell proliferation [85]. CDK5 depletion or inhibition decreases VEGF protein levels and cell proliferation in GH3 cells. CDK5 also enhances the migration and invasion of prolactin pituitary adenomas, predominantly by phosphorylation of kinase insert domain receptor (KDR, also known as VEGFR2) at S229 (Table 2) [86] (Fig. 5B). KDR S229 phosphorylation contributes to the membrane trafficking of functional KDR to cell surfaces [86]. Both inhibition and depletion of CDK5 suppressed cell migration, cell invasion and KDR pS-229 levels in pituitary cells.

### CDK5 acts as an oncogene in Lung cancer

Lung cancer is the second most diagnosed cancer in the US and accounts for ~ 25% of all cancer-related deaths. Lung cancers are classified into small cell lung cancer (SCLC) (~ 15%) and non-small cell lung cancer (NSCLC) (remaining 85%). SCLCs are significantly more aggressive than NSCLCs. CDK5 regulates the prognosis of both SCLCs and NSCLCs, and its overexpression is associated with several clinicopathological factors and poor patient survival (Table 1) [87, 88].

**Fig. 6:**
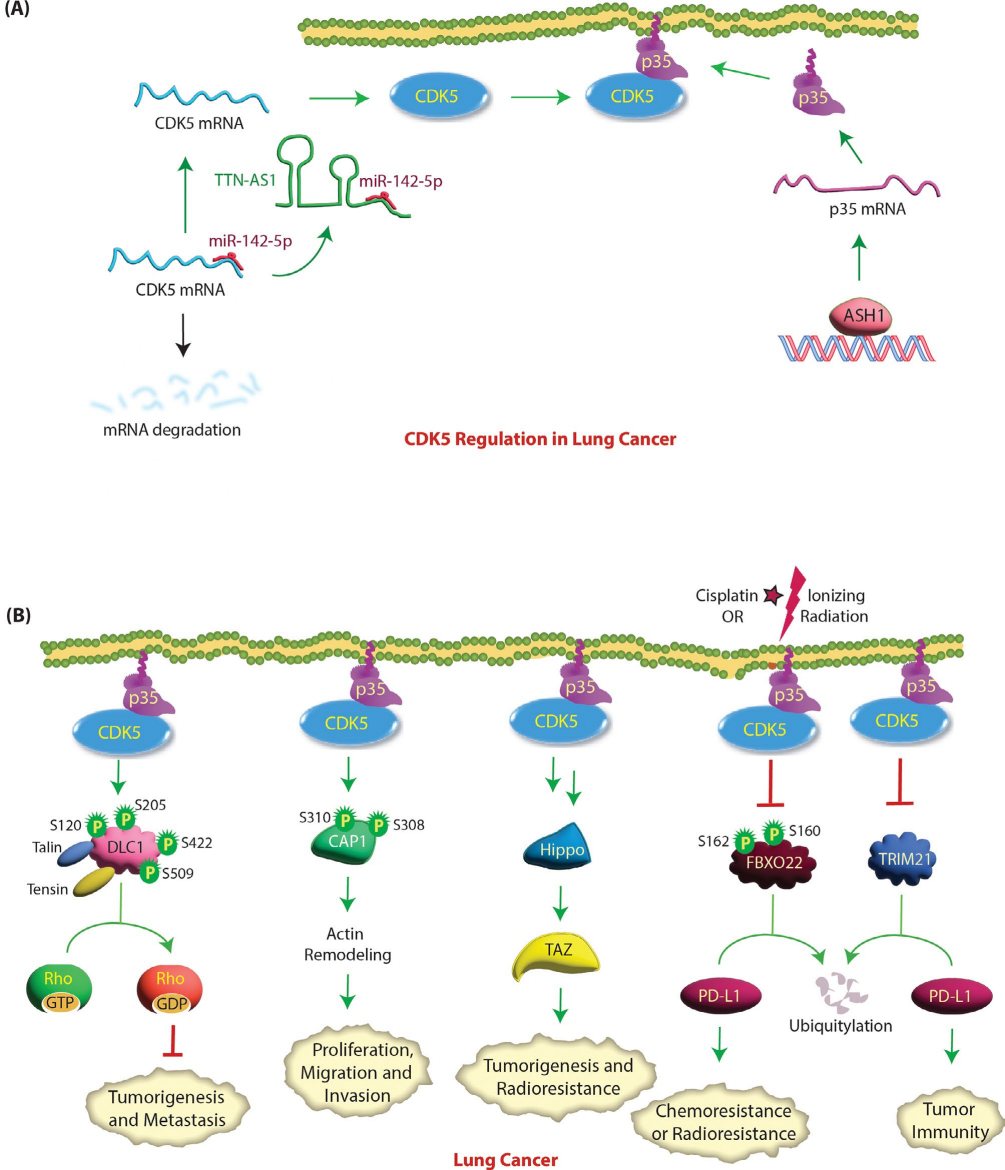
**(A) Molecular mechanisms of CDK5 regulation in lung cancer.** Achaete-scute homolog 1 (ASH1) increases the transcription of p35 to activate CDK5, resulting in increased migration and invasion. miR-142-5p represses CDK5 expression by binding to its 3’-UTR. In lung cancer, TTN-AS1 lncRNA sponges miR-142-5p to upregulate CDK5 transcription. **(B) CDK5 promotes lung tumorigenesis, metastasis, radioresistance and chemoresistance.** Active CDK5 phosphorylates four serine residues in the N-terminal half of DLC1. The phosphorylation of DLC1 by CDK5 changes DLC1 from a closed conformation to an open conformation, which facilitates strong talin and tensin binding and its localization to focal adhesion. This activates the Rho-GAP activity of DLC1, which inactivates Rho GTPases, thereby inhibiting cell migration and tumor growth. Cyclase-associated protein 1 (CAP1) plays an important role in cell movement and morphological changes by acting synergistically with cofilin to regulate cytoskeleton movement. CDK5 knockdown decreased CAP1 phosphorylation followed by a reduction in proliferation and migration, suggesting that CDK5 is an upstream regulator of CAP1. Ionizing radiation or cisplatin treatment induces phosphorylation of FBXO22 at S160 or S162, which activates it, leading to PD-L1 ubiquitylation and causing increased sensitivity to DNA damaging agents. CDK5 inhibition enhances FBXO22 levels resulting in PD-L1 degradation and increased sensitivity to DNA damage. Similarly, CDK5 inhibition causes PD-L1 degradation via TRIM21 resulting in suppressed tumor growth and antitumor immunity. CDK5 activates the Hippo-TAZ signaling pathway, leading to oncogenesis and radioresistance in lung cancer

TTN-AS1, a tumor-promoting lncRNA, increases CDK5 expression in lung adenocarcinoma (LUAD). TTN-AS1 is upregulated in LUAD tissues and associated with TNM stage, lymph node metastasis and poor postoperative prognosis. TTN-AS1 depletion significantly inhibits the proliferation and invasion ability of LUAD cells. TTN-AS1 functions as a competing endogenous RNA by sponging miR-142-5p to upregulate CDK5 in LUAD [89] (Fig. 6A). miR-142-5p represses CDK5 expression by binding its 3’-UTR. In turn, CDK5 promotes EMT, invasion and metastasis in LUAD [89]. Achaete-scute homolog-1 (hASH1 aka human ASH1), a basic helix-loop-helix transcription factor, also acts as an upstream regulator of CDK5 activity in lung cancer. hASH1 promotes the transcription of the CDK5 activator p35 [90], resulting in enhanced proliferation and migration. Silencing ASH1 resulted in decreased p35 expression and reduced migration and invasion (Fig. 6A).

Several downstream targets of CDK5 have been identified in lung cancer. In NSCLC, CDK5 triggers cyclase-associated protein 1 (CAP1) phosphorylation at S308 and S310 (human CAP1 numbering), resulting in actin remodeling [91]. CAP1 phosphorylation at these sites promotes lung cancer tumorigenesis and EMT in vivo [92]. Zeng et al. reported the existence of crosstalk between CDK5 and the Hippo signaling pathway in lung cancer [93]. CDK5 drives lung cancer radioresistance mainly by activating Hippo-TAZ signaling; however, the precise molecular mechanism was not delineated (Fig. 6B).

Programmed cell death ligand 1 (PD-L1) is an immune checkpoint protein that is often overexpressed on tumor cells, including lung cancer cells. It binds to programmed cell death protein 1 (PD-1) on T cells and inhibits CD8 T-cell activity, allowing cancer cells to evade immune elimination. De et al. reported that high levels of FBXO22 (a ubiquitin E3 ligase) sensitize NSCLC cells to cisplatin and ionizing radiation (IR) by degrading PD-L1 [94]. IR induces phosphorylation of FBXO22 at S160 or S162, which is essential for its function; however, the kinase responsible for this event was not identified [94]. The authors further demonstrated that inhibiting or depleting CDK5 increases the level of FBXO22 with a concomitant decrease in PD-L1, leading to increased sensitivity of NSCLC cells to DNA damage (Fig. 6B). Thus, CDK5 promotes chemoresistance and radioresistance by decreasing FBXO22 levels and increasing PD-L1 levels. They predicted that FBXO22 is a substrate of CDK5, although future studies are needed to confirm whether it is a direct or indirect target of CDK5. Similarly, CDK5 inhibition or knockdown initiates TRIM21-triggered degradation of PD-L1, suppressing tumorigenesis and tumor immunity [95] (Fig. 6B).

Paradoxically, CDK5 can also promote the tumor-suppressing functions of Deleted in Liver Cancer 1 (DLC1) in lung cancer [96]. DLC1, a Rho-GTPase activating protein (Rho-GAP), is downregulated in multiple malignancies, including lung cancer [97]. DLC1 promotes focal adhesion turnover, and its Rho-GAP activity inactivates RhoA, -B, and -C [98]. DLC1’s tumor suppressor activity requires its presence at focal adhesions, its Rho-GAP function, and its ability to bind several partners, including tensin, talin, and FAK [99, 100]. CDK5 increases its tumor suppressor activity by phosphorylating it at four serine residues namely S120, S205, S422 and S509 in the N-terminal region next to the Rho-GAP domain, which allows its binding to focal adhesions (talin, tensin) and increases its Rho-GAP activity [96] (Fig. 6B). In this respect, CDK5 acts as a tumor suppressor. The authors reasoned that DLC1 downregulation, a frequent occurrence in NSCLC, could contribute to CDK5’s pro-oncogenic activity. Accordingly, they observed that a substantially higher percentage of poorly differentiated NSCLC has low DLC1 expression coupled with high CDK5 expression compared with well-differentiated NSCLC [96].

### Oncogenic CDK5 in melanoma

Malignant melanoma is the deadliest form of skin cancer, as it is clinically very aggressive and metastatic. CDK5 and its activator p35 are highly expressed in human melanoma cells and tissues (Table 1) [101, 102]. Activation of CDK5 leads to increased cell motility and invasion in melanoma cells both in vitro and in vivo [101, 102]. Pharmacological inhibition or shRNA-mediated knockdown of CDK5 inhibits invasion/migration, colony formation, and anchorage-independent growth of melanoma cells without affecting cell proliferation. Furthermore, CDK5 depletion inhibits lung and liver metastases in an in vivo melanoma model [102].

**Fig. 7:**
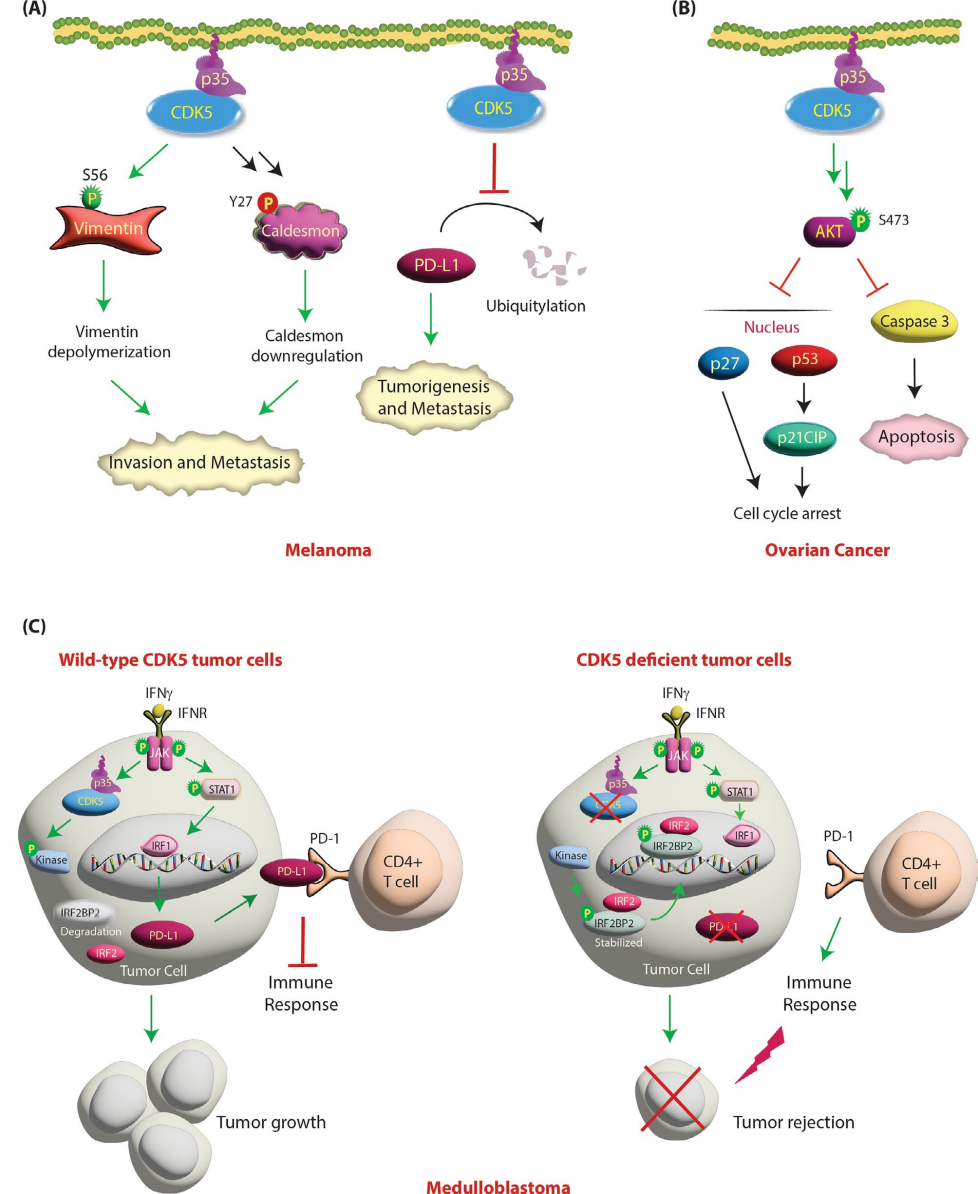
**(A) Regulation of melanoma by CDK5/p35 signaling.** CDK5 regulates cell invasion and metastasis by phosphorylating vimentin in melanoma. CDK5 phosphorylates vimentin at S56, causing disassembly of vimentin filaments and leading to invasion and metastasis. CDK5 activation also leads to phosphorylation of Caldesmon indirectly at Y27, which downregulates it, resulting in increased cell migration. CDK5 inhibits PD-L1 degradation, which ultimately results in enhanced tumorigenesis and metastasis. **(B) CDK5 activates AKT signaling in ovarian cancer.** CDK5 promotes ovarian cancer by upregulating AKT activation and inhibiting caspase-dependent apoptosis. Activation of CDK5 activates AKT, which further promotes ovarian cancer cell proliferation by inhibiting apoptosis and cell cycle arrest by preventing the nuclear translocation of p53 and p27. **(C) CDK5 inhibits immune response in medulloblastomas.** CDK5 inhibition decreases PD-L1 expression to induce antitumor immunity. In wild-type tumor cells, interferon gamma (IFNγ) stimulation activates IRF1-mediated transcription of PD-L1 in tumor cells. PD-L1 binds to programmed cell death 1 (PD-1) on CD4 + T cells, which inhibits the antitumor immune response and results in tumor initiation and growth. Conversely, in CDK5-deficient tumor cells, IRF2 and IRF2BP2 inhibit the induction of PD-L1 transcription by interferon regulatory factor-1 (IRF1) and result in reduced expression of PD-L1. This induces an immune response and results in tumor rejection. Green arrows represent activating pathways

CDK5 was found to regulate melanoma cell invasiveness by directly phosphorylating vimentin [101]. Vimentin, an intermediate filament protein found in mesenchymal cells, plays an essential role in regulating the motility and invasiveness of cancer cells, including melanoma cells. CDK5 phosphorylates vimentin at S56, leading to its depolymerization and invasion (Table 2) [101] (Fig. 7A). In addition, CDK5 affects melanoma invasiveness and migration through regulation of the actin- and calmodulin-binding protein caldesmon [102]. CDK5 inactivation leads to upregulation and dephosphorylation of caldesmon at Y27. As CDK5 does not phosphorylate tyrosine residues, the impact on Y27 appears to be indirect, although the exact mechanism was not elucidated. The authors concluded that CDK5 promotes invasion in vitro and in vivo by facilitating caldesmon phosphorylation via an unknown kinase causing its downregulation.

A central role of CDK5 in antitumor immunity was recently reported by Dorand et al. [103] in subcutaneous medulloblastoma tumor models, which prompted Deng et al. to knock out CDK5 in melanoma cells. As expected, CDK5 depletion elicited a strong T-cell-mediated immune response by downregulating PD-L1, resulting in suppressed 4T1 tumor growth and inhibited lung metastasis [104] (Fig. 7A).

### Role of CDK5 in ovarian cancer

High CDK5 expression is associated with shorter progression-free survival in ovarian cancer (Table 1) [105]. Furthermore, increased expression of CDK5, especially cytoplasmic expression in human ovarian cancers, leads to poor cell survival [106]. CDK5 knockdown inhibits AKT phosphorylation, leading to cell cycle arrest and apoptosis, and this effect is further enhanced by paclitaxel treatment. Mechanistically, G1 cell cycle arrest and apoptotic induction by CDK5 knockdown is due to nuclear translocation of p53 and p27 (KIP1), as well as p53-mediated transcriptional induction of p21CIP1 [106] (Fig. 7B).

### Role of CDK5 in medulloblastoma

Medulloblastoma is the most common pediatric brain malignancy and frequently arises in the cerebellum. Medulloblastoma cell lines and clinical specimens express CDK5, p35 and p39 (Table 1) [103]. Dorand et al. uncovered that CDK5 and PD-L1 expression co-occur in human tumors, which prompted them to investigate their relationship at the molecular level. Treatment of medulloblastoma cell lines with interferon γ (IFNγ) has two major consequences, both of which coordinate to suppress immune evasion by T cells. First, IFNγ treatment increases p35 levels via JAK kinases, resulting in increased CDK5 kinase activity. Second, IFNγ is also the most potent inducer of PD-L1 [107]. Upon IFNγ stimulation, JAK kinases phosphorylate STAT1, causing its activation. Active STAT1 upregulates IRF1, which increases the transcription of PD-L1, permitting tumor cells to evade detection by T cells in vivo. Simultaneously, active CDK5 potentiates PD-L1 transcription by degrading IRF2BP2. CDK5 phosphorylates an unknown kinase, inhibiting its activity, which results in IRF2BP2 degradation. IFNγ-induced IRF1-driven transcription of PD-L1 is inhibited by interferon regulatory factor-2 (IRF2) and its corepressor IRF2BP2. Accordingly, CDK5 depletion or inhibition led to reduced PD-L1 expression in response to IFNγ stimulation with a concomitant increase in the levels and stability of IRF2 and IRF2BP2. Furthermore, diminished PD-L1 expression increased the numbers of CD4 + tumor-infiltrating lymphocytes, resulting in robust CD4 + T-cell-mediated tumor rejection [103]. As CDK5 downregulates FBXO22, which in turn increases PD-L1 levels in NSCLC, this mechanism may also occur in medulloblastoma. Future studies are needed to uncover this mechanism (Fig. 7C).

### Role of CDK5 in head and neck squamous cell carcinoma (HNSCC)

**Fig. 8:**
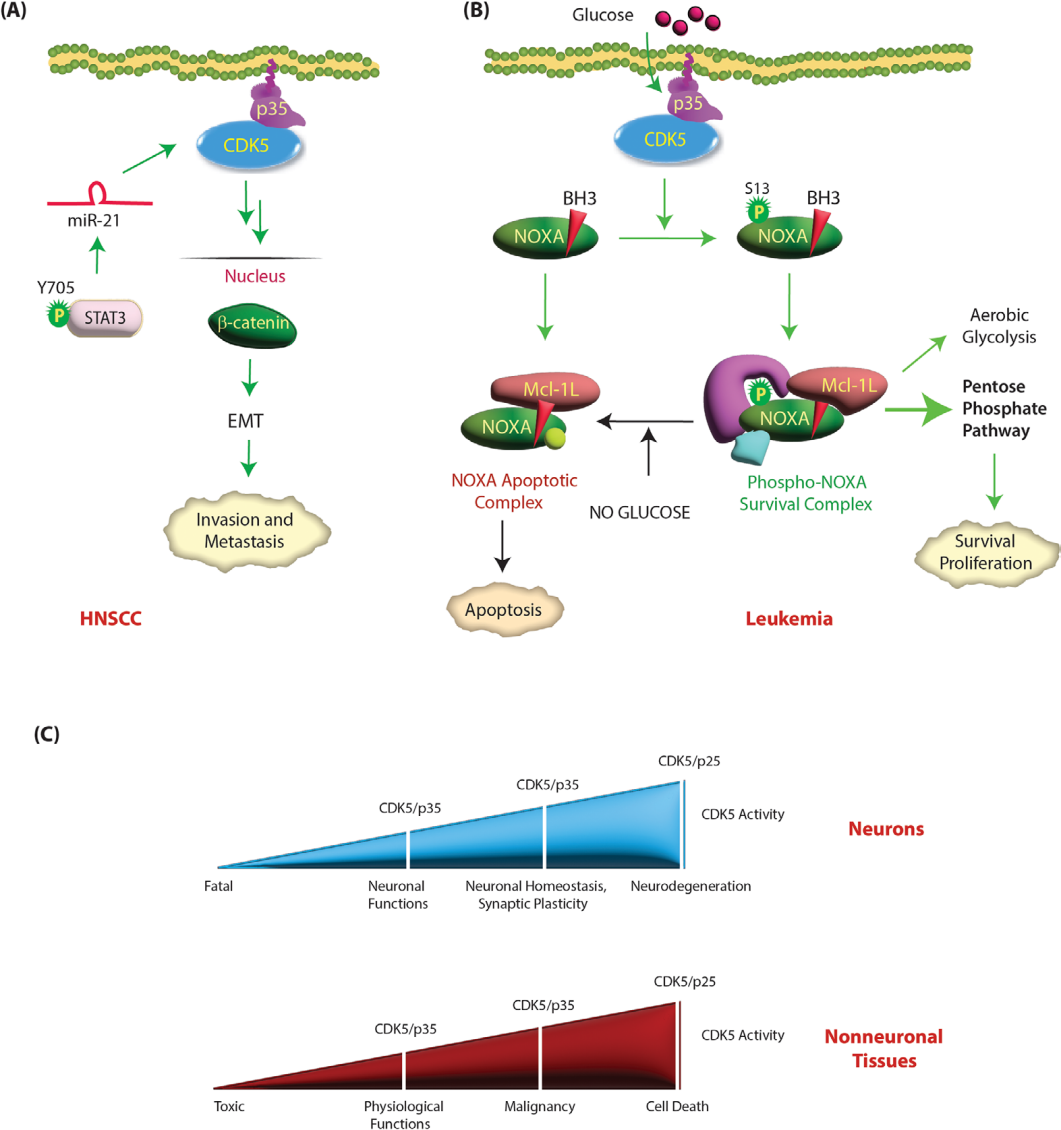
**(A) CDK5 acts as an oncogene in HNSCC.** Tyrosine-phosphorylated active STAT3 induces the expression of miR-21, which in turn upregulates CDK5 and p35. CDK5/p35 in turn increase the nuclear translocation of β-catenin, leading to EMT, invasion and metastasis. Green circle shows activating phosphorylation event. **(B) CDK5 promotes survival in leukemia cells.** BH3-only protein NOXA plays dual roles in proliferating hematopoietic cancer. In the presence of glucose, NOXA is phosphorylated by CDK5 at S13 and is sequestered in a cytosolic complex that imparts a metabolic (growth and survival) advantage. Both glucose deprivation and CDK5 inhibition promote apoptosis by dephosphorylating NOXA. **(C) CDK5 acts as a molecular rheostat in neuronal and nonneuronal tissues**. Upregulation of CDK5 and p35 in neurons and nonneuronal tissues has distinct consequences. While overexpression of CDK5 and/or p35 in neurons is neuroprotective, it is oncogenic in nonneuronal tissues. Nevertheless, emerging evidence suggests that hyperactivation of CDK5 by p25 formation is toxic in both neurodegenerative diseases and cancer

Aberrant overexpression of CDK5 significantly induces tumor cell motility and EMT in HNSCC (Table 1) [108]. Sun et al. showed that overexpression of miR-21/CDK5 was associated with EMT and lymph node metastasis in HNSCC. Mechanistically, levels of active STAT3 (phosphorylated at Y705) are increased, which in turn increases miR-21 levels. miR-21 promotes the expression of CDK5 and p35, which results in nuclear translocation of β-catenin, resulting in EMT. miR-21 depletion impaired HNSCC migration, invasion and EMT by downregulating CDK5/p35 (Fig. 8A) [108].

### CDK5 promotes survival in leukemia

So et al. observed that CDK5, p35 and Cyclin I mRNA levels are increased in acute myeloid leukemia (AML) patients in The Cancer Genome Atlas dataset (Table 1) [109]. Importantly, CDK5 is also frequently deleted in a subset of AML patients, although the prognostic significance of this event is not fully understood [110]. Levacque et al. analyzed multiple microarray datasets consisting of 2880 leukemia patients, and concluded that CDK5 mRNAs were frequently downregulated in leukemia, although some patient specimens also showed CDK5 upregulation [111]. The proteins levels of CDK5 and its activators have not been analyzed in leukemia specimens to date.

CDK5 promotes growth and survival by phosphorylating BH3-only protein NOXA in hematopoietic cells. NOXA is constitutively expressed in proliferating cells of the hematopoietic lineage. NOXA interacts almost exclusively with the prosurvival Bcl-2 protein Mcl-1L, inactivating its survival function and thereby promoting apoptosis [112] (Fig. 8B). However, in the presence of glucose, NOXA is phosphorylated at S13 by CDK5, which promotes cytosolic sequestration and inhibits apoptosis. Furthermore, S13-phosphorylated NOXA stimulates glucose consumption presumably via the pentose phosphate pathway instead of glycolysis. Thus, proapoptotic NOXA promotes growth in hematopoietic cancers by CDK5 [112]. Accordingly, both CDK5 inhibition and glucose deprivation trigger apoptosis by dephosphorylating NOXA. Therefore, CDK5-dependent phosphorylation acts as a glucose-sensitive switch that allows NOXA to play either a growth-promoting or proapoptotic role in leukemia (Fig. 8B).

So et al. demonstrated that CDK5 or CCNI loss sensitized cells to mitochondrial enzyme dihydroorotate dehydrogenase (DHODH) inhibition, while p35 knockdown did not, indicating that CDK5 likely partners with CCNI in AML [109]. Nevertheless, the exact mechanism by which CDK5 acts as a synthetic lethal partner for DHOHD remains unclear. Future studies are required to fully uncover the specific roles of CDK5 and its activators in this disease.

### Role of CDK5 in multiple myeloma

Multiple myeloma (MM) is an incurable plasma cell malignancy of bone marrow. Increased expression of CDK5 is observed in both primary MM cells and MM cell lines compared to normal bone marrow mononuclear cells (BMMCs) (Table 1) [111, 113]. High expression of CDK5 is associated with poor prognosis and bortezomib resistance in MM patients. CDK5 knockdown suppresses the viability of MM cells and sensitizes them to bortezomib, a proteasome inhibitor that generates considerable clinical response in newly diagnosed as well as advanced multiple myeloma patients [113]. The exact mechanism of bortezomib action in MM is still unidentified. However, it has been reported that bortezomib reduces NFκB activity and induces the unfolded protein response, ER stress and immune sensitization [113]. Bortezomib also inhibits the transcription of many DNA repair enzymes, including those involved in nonhomologous end joining, mismatch repair, base excision repair and nucleotide excision repair [114]. As CDK5 acts on the G_2_/M checkpoint to prevent passage into mitosis upon DNA damage [115], a lack of CDK5 allows unregulated mitotic entry that increases genomic instability and, thus, apoptosis.

Eph receptor A4 (EphA4) is overexpressed in several tumors and promotes the proliferation of multiple myeloma cells. EphA4 binds CDK5 and increases its expression, leading to enhanced AKT activation and cell adhesion-mediated drug resistance in multiple myeloma [116].

## Conclusions

CDK5 has emerged as a critical player in both cancer and neurodegenerative diseases. Nevertheless, some critical differences govern CDK5 deregulation modes in these disparate diseases. While CDK5 and/or p35 upregulation in neurons promotes healthy neuronal functions, their upregulation in cancer promotes highly aggressive phenotypes (Fig. 8C). Similarly, CDK5 hyperactivation upon neurotoxic insults causes mitochondrial dysfunction leading to cell death in AD [47], whereas in breast cancer, loss of CDK5 has the same impact.

In neurodegenerative diseases, p25 formation is crucial for CDK5 deregulation due to its long half-life and by mislocalizing active CDK5 to the cytoplasm and nucleus. In contrast, p25’s contribution to cancer progression is less understood. In pancreatic cancer, K-Ras leads to p25 formation through an unknown mechanism. However, as the vast majority of pancreatic tumors overexpress CDK5, p35 and p39, the contribution of p25 to disease pathogenesis remains unclear. Bibb et al. created an inducible p25 MTC mouse model in which proliferation was triggered upon p25 expression in C-cells and halted by interrupting p25 overexpression [34]. As both p35 and p25 are enriched in MTC tissues, the exact contribution of p25 is still not well understood. Lin et al. indeed demonstrated that p35 cleavage does not contribute to CDK5 activity-dependent cell proliferation in MTC, as neither Cdk5 activity nor MTC cell growth was altered by the inhibition of p35 cleavage [62].

In neurons, an initial increase in p25 levels promotes synaptic plasticity; however, an increase beyond a certain threshold leads to neurodegeneration. We believe a similar mechanism exists in cancer, where a moderate increase in p25 may promote oncogenicity, but high levels of p25 are fatal. A few studies have indeed shown that hyperactivation of CDK5 by inducing p25 formation causes apoptosis in cancer [69, 117]. In this respect, CDK5 hyperactivation in cancer mimics neurodegenerative diseases where CDK5 deregulation via p25 is toxic. Thus, CDK5 acts as a molecular rheostat, with different activity levels eliciting distinct functional outcomes. Accordingly, low CDK5 activity is toxic, medium activity supports a variety of physiological functions, high activity promotes malignancy, and very high activity causes cell death (Fig. 8C). Thus, hyperactivating CDK5 by p25 formation in cancer may be an effective approach to promote cell death. Future studies are needed to precisely manipulate p25 levels in vivo to maximize its potential as an anti-oncogenic therapy while avoiding any oncogenic effects.

An increase in CDK5 levels or activity is frequently associated with highly aggressive oncogenic phenotypes and a poor prognosis. However, in these cancers, CDK5 also inhibits oncogenic pathways through a few substrates. CDK5 activates tumor-suppressive pathways in pancreatic cancer via EZH2 degradation and in NSCLC by DLC1 activation. As DLC1 is often downregulated in lung cancer, CDK5-mediated DLC1 activation may have a limited impact. Likewise, EZH2 is predominantly nuclear in lung cancer. Therefore, CDK5-triggered degradation of cytoplasmic EZH2 may also have little effect. Uniquely in gastric cancer, CDK5 acts as a tumor suppressor. This has been attributed to its nuclear localization, allowing it access to distinct substrates. Although CDK5-EZH2 crosstalk has not been analyzed in gastric cancer, it is possible that CDK5 degrades nuclear EZH2 in gastric cancer, triggering tumor-suppressive pathways. Finally, as both EZH2 and CDK5 have independently been shown to be critical for NEPC progression, it is likely that they reside in different compartments where each promotes specific oncogenic pathways. Future studies are needed to uncover their potential crosstalk in NEPC.

Most importantly, analysis of CDK5’s substrates and pathways in sixteen different cancers suggests that CDK5’s behavior as an oncogene or an anti-oncogene depends upon its subcellular localization (Table 1). Based on the findings, it appears that cytoplasmic CDK5 is oncogenic, and nuclear CDK5 is tumor-suppressive. Thus, directing CDK5 to a different location may be an effective way to reverse tumorigenesis than inhibiting CDK5 clinically. Furthermore, the use of small molecules to control CDK5’s subcellular localization could be a promising approach to treating cancer. Equally importantly, it is critical to analyze the subcellular localization of CDK5 activators in each cancer. The subcellular location of the activator may be the main determinant of CDK5’s oncogenic or anti-oncogenic behaviors in some cancer cells, in which CDK5 resides both in the cytoplasm and nuclear compartments.

Finally, more than two dozen direct substrates of CDK5 have been identified in cancer to date (Table 2). Some of these direct and indirect targets are shared in different cancers, including PD-L1 (melanoma, medulloblastoma and lung cancer), Rb (prostate and MTC), STAT3 (PCa and MTC) and talin (breast and prostate) (Table 2). CDK5 was shown to transcriptionally upregulate PD-L1 in medulloblastoma and stabilize it in lung cancer, both allowing tumor immunity. While CDK5 should certainly have distinct tissue-specific substrates, regulatory mechanisms for many of its common substrates may be similar in different tissues, analysis of which should aid in identifying critical signaling nodes, particularly for each tissue-specific cancer. Furthermore, identification of these substrates provides further therapeutic intervention points for developing effective therapies either alone or in combination with CDK5. Finally, CDK5’s role as an oncogene or a tumor suppressor is defined by its access to distinct substrates. As a result, directing it to a different subcellular location using small molecules could be a promising approach to treat cancer, analogous to neuronal death triggered by nuclear CDK5. Alternatively, exporting tumor-suppressive nuclear substrates to the cytoplasm may also be an effective strategy to combat CDK5-induced oncogenicity.

## Data Availability

Not applicable.

## Abbreviations

CDKs: Cyclin-dependent kinases
CDK5: Cyclin-dependent kinase-5
GSTP1: Glutathione S-transferase P1
EMT: Epithelial Mesenchymal Transition
MTC: Medullary thyroid carcinoma
ADD1: Adducin1
PPARγ: Peroxisome proliferator-activated receptorγ
PCa: Prostate cancer
PDX: Patient-derived xenograft
PDAC: Pancreatic ductal adenocarcinoma
Ral GEFs: Ral guanine nucleotide-exchange factors
EZH2: Enhancer of zeste homolog
GBM: Glioblastoma
PIKE: A-Phosphatidylinositol 3 kinase enhancer-A
CRMP2: Collapsin response mediator protein 2
ACSS2: Acetyl-CoA synthetase 2
OGT: O-GlcNAc transferase
DYRK1A: Dual-specificity tyrosine phosphorylation-regulated kinase 1 A
HCC: Hepatocellular carcinoma
PRMT1: Protein arginine methyltransferase 1
SCLCs: Small cell lung cancer
NSCLCs: Non-small cell lung cancer
LUAD: Lung adenocarcinoma
hASH1: Achaete-scute homolog-1
CAP1: Adenylate cyclase associated protein 1
PD: L1-Programmed cell death ligand 1
IFNγ: Interferonγ
HNSCC: Head and neck squamous cell carcinoma
MM: Multiple myeloma
EphA4: Eph receptor A4
ATM: Ataxia Telangiectasia Mutated
DLC: 1-Deleted in Liver Cancer 1
FAK: Focal adhesion kinase
PIPKIγ90: Phosphatidylinositol 4-phosphate 5-kinase type I γ90
ERK: Extracellular signal-regulated kinase
TRIM59: Tripartite motif-containing 59
RPA32: Replication protein A
ESRP1: Epithelial Splicing Regulatory Protein 1
DRP1: Dynamin-related protein 1
TIGAR: TP53 induced-glycolysis regulatory phosphatase
CK2: Casein kinase 2
mPTP: Mitochondrial permeability transition pore
AR: Androgen receptor
TNBC: Triple-negative breast cancer
STAT: Signal transducer and activator of transcription
